# An imputation approach using subdistribution weights for deep survival analysis with competing events

**DOI:** 10.1038/s41598-022-07828-7

**Published:** 2022-03-09

**Authors:** Shekoufeh Gorgi Zadeh, Charlotte Behning, Matthias Schmid

**Affiliations:** Department of Medical Biometry, Informatics and Epidemiology, Faculty of Medicine, University of Bonn, Sigmund-Freud-Str: 25, D-53127 Bonn, Germany

**Keywords:** Machine learning, Statistical methods

## Abstract

With the popularity of deep neural networks (DNNs) in recent years, many researchers have proposed DNNs for the analysis of survival data (time-to-event data). These networks learn the distribution of survival times directly from the predictor variables without making strong assumptions on the underlying stochastic process. In survival analysis, it is common to observe several types of events, also called competing events. The occurrences of these competing events are usually not independent of one another and have to be incorporated in the modeling process in addition to censoring. In classical survival analysis, a popular method to incorporate competing events is the subdistribution hazard model, which is usually fitted using weighted Cox regression. In the DNN framework, only few architectures have been proposed to model the distribution of time to a specific event in a competing events situation. These architectures are characterized by a separate subnetwork/pathway per event, leading to large networks with huge amounts of parameters that may become difficult to train. In this work, we propose a novel imputation strategy for data preprocessing that incorporates weights derived from a time-discrete version of the classical subdistribution hazard model. With this, it is no longer necessary to add multiple subnetworks to the DNN to handle competing events. Our experiments on synthetic and real-world datasets show that DNNs with multiple subnetworks per event can simply be replaced by a DNN designed for a single-event analysis without loss in accuracy.

## Introduction

In the recent years deep networks have become the state-of-the-art method in various applications, for instance in object detection ^1^, image captioning ^2^, image classification^3,4^, speech recognition ^5^, and many other areas. One key advantage of deep neural networks is their capacity to learn specific intermediate representations/features of the data in a hierarchical manner^6^ in order to create a mapping from the input predictor variables onto the outcome. In addition to other novel machine learning methods developed for survival analysis^7^, recently, there has been a growing interest in using deep neural networks for this purpose, see for example, the works by Giunchiglia et al.^8^, Lee et al.^9^, Zafar Nezhad et al.^10^ and many others^11–16^.

In survival analysis the outcome is usually defined by the time duration until one or more events occur^17^. For instance in the medical field this event could be recurrence of a disease or patient’s death after an intervention. A multitude of examples can e.g. be found in the work by Lee et al.^18^. Since survival data (also called *time-to-event* data) are collected over time, they are often subject to right censoring, which means that the event times of some instances are only known up to a minimum survival time. The real event times of these instances remain unknown as they are no longer observed beyond the time of censoring. Often, right censoring occurs when patients drop out of a study or when patients have not experienced any event before study end.

Many observational studies track more than one event. Often these so-called *competing events* do not occur independently, and therefore require to be analyzed together in order to avoid bias. For instance, in the CRASH-2 trial^19^, which is a large randomized study on hospital death in adult trauma patients, there are multiple recorded causes of death throughout the study. The causes include death due to bleeding, head injury, multi-organ failure and others. Obviously, the occurrences of these causes are not independent. More examples on competing risks data can be found in the works by Lau et al.^20^ and Austin et al.^21^.

For modeling the time span until a specific event of type $j\in \{1,\ldots ,J\}$ occurs, multiple approaches have been proposed. For example, Prentice et al.^22^ model the *cause-specific hazard functions* of each event separately as $\xi _{j}(t|x)=\lim _{\Delta t\longrightarrow 0}\{P(t \le T < t+\Delta t,\epsilon =j \, | \, T \ge t,x)/\Delta t\}$, where $x=(x_1,\ldots ,x_p)^T$ is the vector of time-constant predictor variables and $\epsilon$ is a random variable indicating the type of the event that occurs at the first observed event time *T*. In their approach, a separate model is used for each $\xi _j$, treating the individuals that experience any of the respective competing events as censored. Another approach, on which the methods considered in this paper are based, is the subdistribution hazard model by Fine and Gray^23^. This approach aims at modeling the *cumulative incidence functions* $F_j (t|x) = P (T \le t, \epsilon = j \, | \, x)$. For any event *j* of interest, the model considers a *subdistribution hazard* function $\lambda _{j}(t|x)=\lim _{\Delta t\longrightarrow 0}\{P(t \le \vartheta _j < t+\Delta t \, | \, \vartheta _j \ge t,x)/\Delta t\}$, where $\vartheta _j$ is the “subdistribution time” defined by $\vartheta _j = T$ if $\epsilon = j$ and $\vartheta _j = \infty$ otherwise. Thus, $\vartheta _j$ corresponds to the time to the occurrence of a type-*j* event, assuming that such an event can never be observed (i.e. $\vartheta _j = \infty$) if a competing event occurs first. It can be shown^23^ that specifying a regression model for $\lambda _{j}(t|x)$ allows for modeling cumulative incidences of type-*j* events without having to model the hazard functions of the other events. Thus, only one subdistribution hazard model is required if the interest is in the cumulative incidence function of the type-*j* event. This is unlike cause-specific hazards modeling, where all $\xi _1 , \ldots , \xi _J$ need to be considered together to calculate cumulative incidence probabilities.

To analyze competing events data using deep neural networks, Lee et al.^9^ proposed the DeepHit network that directly learns the distribution of survival times for an event of interest while at the same time handling the competing events. In their architecture, a separate subnetwork is added for each competing event. Similarly, Gupta et al.^11^ use separate subnetworks per event. In another work, Nagpal et al.^24^ proposed a Deep Survival Machine (DSM) to learn a mixture of parametric distributions (e.g. Weibull or log-normal) for estimating the conditional survival function $S(t|x) = P(T > t |x)$. Again, in this model an additional set of parameters is added to describe the event distribution for each competing risk.

In this work, instead of extending a network’s architecture by multiple subnetworks to handle competing events, we follow the approach by Fine and Gray and propose to employ deep network architectures for a *single* event of interest^8,25–27^. To incorporate competing events, our method works on input data that have been preprocessed using an imputation strategy based on subdistribution weights (see Methods section for details). As will be demonstrated, this strategy allows analysts to benefit from the advantages of existing single-event implementations for time-to-event data (particularly, from much simpler architectures with smaller sets of parameters) while being able to avoid a possible bias caused by ignoring competing events. In our experiments on simulated and real-world datasets, we show that approximately the same performance can be gained without the need for specifying a complex network architecture with multiple event-specific parameter sets.

The key contributions of this work are: (1) We propose a novel preprocessing strategy for deep survival networks that enables a valid analysis of competing-risks data, even if the respective network architecture was originally designed to handle one event only. (2) We demonstrate the feasibility of our approach by comparing two variants of the DeepHit architecture. Specifically, we compare a DeepHit model with *two* subnetworks (designed to analyze the original input data with two competing events) to a DeepHit model with only *one* subnetwork (designed to analyze one event of interest and based on a modified input data set that was preprocessed using our imputation method). (3) Using simulations, we analyze the behavior of deep survival architectures that are designed to analyze one event of interest. Specifically, we demonstrate that these architectures perform better (in terms of both calibration and discrimination) when the proposed preprocessing strategy is applied than when the original input data (treating observations with a competing event as censored) are used.

## Methods

### Notations and definitions

To be able to use single-event DNN architectures like DeepSurv^25^, SurvivalNet^26^, RNN-Surv^8^ and DRSA^27^, continuous survival and censoring times have to be grouped. To this end, we define time intervals $$[0, a_1), [a_1, a_2), . . . , [a_{k-1},\infty )$$, where *k* is a natural number. Further denote by $$T_i \in \{1, . . . , k\}$$ and $$C_i \in \{1, . . . , k\}$$ the resulting discrete event and censoring times, respectively, of an individual contained in an i.i.d. sample of size *n*, $$i = 1, \ldots , n$$. In this definition, $$T_i=t$$ means that the event has happened in time interval $$[a_{t-1}, a_t)$$. It is assumed that $$T_i$$ and $$C_i$$ are independent random variables (“random censoring”). Furthermore, it is assumed that the censoring time does not depend on the parameters used to model the event time, i.e. the censoring mechanism is “non-informative” for $$T_i$$^22,28^. For right-censored data, the observed time is defined by $$\tilde{T}_i = \text {min}(T_i,C_i)$$, i.e. $$\tilde{T}_i$$ corresponds to true event time if $$T_i \le C_i$$, and to the censoring time otherwise. The random variable $$\Delta _i := I(T_i \le C_i)$$ indicates whether $$\tilde{T}_i$$ is right-censored $$(\Delta _i = 0)$$ or not $$(\Delta _i = 1)$$. In addition to the event of interest (defined without loss of generality by $$j=1$$), we assume that each individual can experience one out of $$J-1$$ competing events, $$j \in \{2,\ldots , J\}$$. The type of event that the *i*-th individual experiences at $$T_i$$ is represented by the random variable $$\epsilon _i \in \{1, . . . , J\}$$^29^. The values of the predictor variables of the *i*-th individual are denoted by $$x_i = (x_{i1},\ldots ,x_{ip})^T$$. Analogous to the works by Fine and Gray^23^ and Berger et al.^30^, we are interested in modeling the cumulative incidence function $$F_1(t |x) = P(T \le t, \epsilon = 1 \, | \, x)$$ of a type-1 event using the subdistribution hazard approach described above. To fit their proposed models, both Fine & Gray and Berger et al. considered the optimization of *weighted* versions of the underlying partial and binomial log-likelihood functions. While these techniques turn out to be highly effective when fitting parametric models to sets of lower-dimensional data, it is challenging to adapt them to learning tasks involving deep survival models. Specifically, the method by Fine & Gray relies on a continuous time scale and does not apply directly to the discrete (grouped) event times specified above. On the other hand, the method by Berger et al., which extends the Fine & Gray method to discrete event times, requires the input data to be “augmented” to up to $$n \cdot k$$ instances, implying a potentially huge increase in dimension. Clearly, this approach is not feasible for deep learning tasks, which typically rely on large values of *n*. We propose to address the aforementioned challenges by specifying a preprocessing strategy that operates directly on the discrete event times, while at the same time preserving the dimension of the input data.

### Imputation strategy

In this section we describe the imputation strategy to preprocess the time-discrete input data. The aim is to modify the data such that it is possible to obtain valid estimates of the cumulative incidence function $F_1(t|x)$ by training a single-event DNN. As outlined in the Introduction section, training could be based on the specification of a subdistribution time $\vartheta \equiv \vartheta _1$, which could be subsequently used to learn a single-event DNN with input data $(\min (\vartheta _i, C_i), I(\vartheta _i \le C_i), x_i^\top )$, $i=1,\ldots , n$. A problem of this strategy is that it cannot be readily applied in practice, as the aforementioned input data are partly unknown. We therefore propose to apply additional preprocessing steps to the available input data. The details are as follows:

First, consider those individuals *i* with $\Delta _i \epsilon _i \in \{ 0,1\}$. Clearly, it is not necessary to preprocess the input data of these individuals, since both $\min (\vartheta _i, C_i) =\tilde{T}_i$ and $I(\vartheta _i \le C_i) = \Delta _i$ are known in these cases. Next, consider those individuals who experience a competing event first, i.e. $\Delta _i \epsilon _i > 1$. For these individuals $\vartheta _i = \infty$, so that $I(\vartheta _i \le C_i) = 0$ is known. However, $\min (\vartheta _i, C_i) = \min (\infty , C_i) = C_i$ is unknown in these cases due to the fact that the value of the censoring time $C_i$ is unobserved.

**Figure 1:**
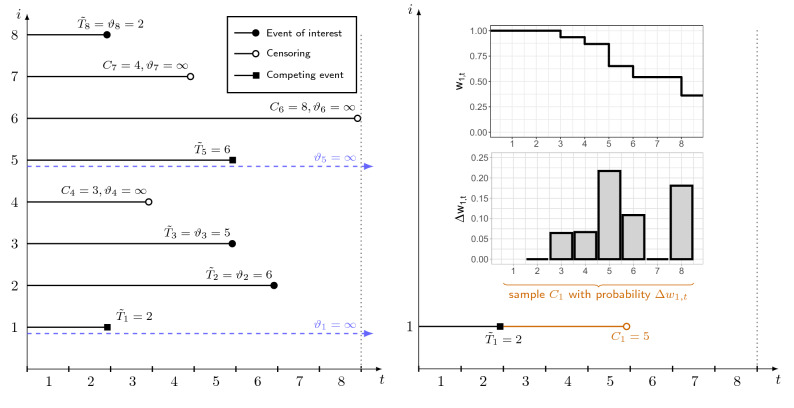
Illustration of the imputation strategy. The left panel presents the subdistribution times of eight randomly sampled individuals. Individuals 1 and 5 experienced the competing event first, implying that their censoring times are unobserved (as illustrated by the time span for $i=1$ in the right panel). For these individuals, censoring times are estimated by first calculating estimated subdistribution hazard weights $w_{i,t}$ (see upper right diagram). From that, the weight differences $\Delta w_{i,t}$ are calculated and used to sample censoring times $C_i$, which are in turn used to impute the unobserved values of $C_i = \min (C_i, \vartheta _i)$. Note that the bars in the lower right panel correspond to the heights of the steps in the upper right panel.

The main idea of our approach is, therefore, to impute the missing values of $C_i$ by sampling a censoring time for any individual *i* who experiences a competing event first. Our strategy is as follows:

(i) Following Berger et al.^30^, we first define the set of discrete *subdistribution weights*$u_{it}=I(t\le \min (\vartheta _i, C_i) )$, $i=1,\ldots ,n$, $t=1,\ldots ,k-1$, indicating whether individual *i* is at risk of a type-1 event at time point *t* ($u_{it}=1$) or not ($u_{it}=0$). We further denote by *r*(*t*) the *risk set* of individuals who have neither experienced a type-1 event nor have been censored before *t*. As outlined above, *r*(*t*) is not fully known for individuals who experience a competing event first. These individuals remain at risk beyond $\tilde{T}_i$ until eventually they experience the censoring event.

(ii) In line with Fine & Gray^23^ and Berger et al.^30^, we specify an *estimate* of the subdistribution weights that can be computed from the available data. Denoting this estimate by $w_{it}$, $i=1,\ldots , n$, $t=1, \ldots , k-1$, we set $w_{it}=1$ if $t\le \tilde{T}_i$, knowing that individuals remain at risk (i.e. belong to *r*(*t*)) until $\tilde{T}_i$. For $t > \tilde{T}_i$ and $\Delta _i\epsilon _i > 1$, we estimate $u_{it}$ by the conditional probability of individual *i* being part of *r*(*t*), given knowledge that it is part of $r(\tilde{T}_i)$. This conditional probability can in turn be estimated by

1 $$\begin{aligned} w_{it}:=\frac{\hat{G}(t-1)}{\hat{G}(\tilde{T}_{i}-1)},\quad \tilde{T}_{i}<t\le {k-1}, \end{aligned}$$

where $\hat{G}(t)$ is an estimate of the censoring survival function $G(t) = P(C_i>t)$. For the experiments in this paper, we used the R package *discSurv*^31^, which implements a nonparametric life table estimator to obtain estimates of *G*(*t*).

(iii) In the final step, we use $w_{it}$ to sample estimates of the censoring times of individuals who experience a competing event first. For this, we generate random numbers $\hat{C}_i$ from discrete distributions with supports $(\tilde{T}_{i}+1 ,\ldots , k-1)$ that are defined by $P(\hat{C}_i = t) = \Delta w_{it}$, where $\Delta w_{it} = w_{i t-1}- w_{i t}$. The so-obtained numbers are subsequently used to impute the unobserved values $\min (\vartheta _i, C_i)$. A visualization of the proposed imputation strategy is presented in Fig. 1.

Note that our method bears some similarities to the work by Ruan and Gray^32^, who suggested a multiple imputation approach to model continuous-time survival data in a non-DNN context. The preprocessing strategy proposed here differs from Ruan and Gray^32^ in three aspects: First, Ruan and Gray considered models in continuous time, whereas the DNN architectures considered here operate on a discrete time scale. Accordingly, Ruan and Gray used a conditional Kaplan-Meier estimator to estimate the censoring distribution, implying that the resulting weight differences $\Delta w_{it}$ occur at random time points (whereas we consider fixed [user-specified] interval borders $a_1< a_2< \ldots < a_{k-1}$ to define $\Delta w_{it}$). Second, Ruan and Gray proposed to estimate their quantities of interest (e.g. the parameters of a proportional subdistribution hazard model and/or cumulative incidence functions at selected time points) by applying a multiple imputation strategy. Accordingly, the authors proposed to generate multiple imputed data sets and to average estimates from the respective (multiple) analyses based on the imputed data. This is in contrast to our approach, which assumes that DNN architectures are able to capture the relevant aspects of the data-generating process using a single imputation only. Third, Ruan and Gray mostly focus on semiparametric survival models in a non-machine-learning context (“allowing standard software to be used for the analysis”), whereas the focus of this work is on the nonparametric estimation of cumulative incidence functions using DNN architectures with potentially higher-dimensional predictor spaces.

In the next section we demonstrate that without loss of accuracy, the use of the imputed data simplifies the analysis of competing-risks data by training single-event DNNs.

## Experimental analysis

### DeepHit network

**Figure 2:**
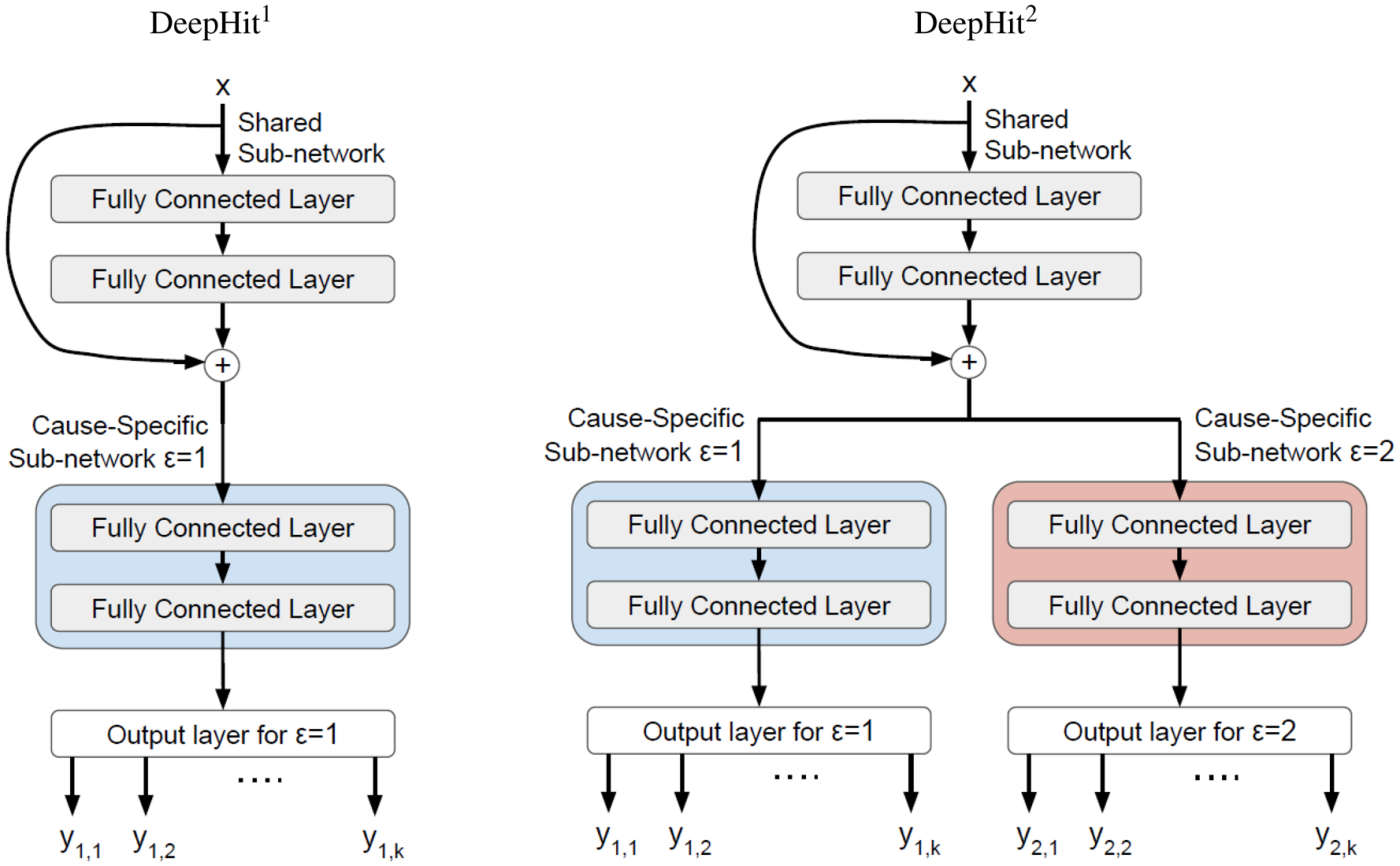
Visualization of the DeepHit$^{1}$ and DeepHit$^{2}$ architectures used in the experiments.

To investigate the effectiveness of the proposed method, we used the DeepHit architecture by Lee et al.^9^. DeepHit is a DNN that allows to have a learnable survival function that maps the predictor variables vector $x_i$ into a probability distribution vector $\mathbf{y} _i=\left[ y_{1,1}, \dots , y_{1,k}, \dots , y_{J,1},\dots , y_{J,k} \right]$. In this vector, element $y_{\epsilon ,t}$ is the estimated probability that instance *i* with predictor variables $x_i$ will experience the $\epsilon$th event at time point *t*. Through non-linear activation functions, DNNs, and in particular DeepHit can learn potentially non-linear, even non-proportional, relationships between the predictor variables and the events^9^. A fully connected layer consists of neurons connected to all neurons in the adjacent layer. Each neuron works as a simple linear classifier ($h=f\left( \sum v_{m}x_{m}\right)$, where *h* is the output, $v_m$ is the network weight, $x_m$ the input from the *m*th neuron in the previous layer, and *f* is the activation function) that receives input from the neurons in the previous layer and sends output to every neuron in the next layer. DeepHit consists of a “shared sub-network” that has two fully connected layers. (Note that in the work by Lee et al.^9^, the authors use one fully connected layer for their experiments. However, empirically we found that using two fully connected layers improves the overall accuracy.) The shared sub-network creates an intermediate representation that is further combined with the input features and passed on to *J* “cause-specific sub-networks”. As recommended by Lee et al.^9^, we used two fully connected layers in each sub-network. The output of each cause-specific sub-network is a vector that estimates the probability of the first hitting time of a specific cause *j* at each time point *t* (see Fig. 2). For training DeepHit, the authors use the log-likelihood of the joint distribution of the first hitting time as well as another loss term to incorporate a mixture of cause-specific ranking loss functions. They also modified the loss to handle right-censored data. In our experiments, we use the same loss term that was used to optimize DeepHit^9^.

To assess the performance of our proposed method we compared three different setups: (1) *New approach using single-event DNN with preprocessed input data:* We trained the DeepHit network with only one subnetwork (see Fig. 2, DeepHit$^{1}$). Instead of the original input data, we used the modified version of the input data (with $T_i$ replaced by $\vartheta _i$), in which the censoring times corresponding to individuals with observed competing events were imputed using the subdistribution weights. (2) *Original DeepHit approach with J subnetworks:* We trained the DeepHit network with a separate cause-specific subnetwork per event (see Fig. 2, DeepHit$^{2}$) (3) *Single-event DNN that ignores competing events:* Similar to the first setup, we train the DeepHit network with only one subnetwork. Instead of replacing $T_i$ by $\vartheta _i$, we ignored the competing events and treated all individuals with an observed competing event as censored (i.e., we treated the observed time to the occurrence of the competing event as the censoring time).

Each experiment was repeated 10 times per dataset in order to reduce the effect of random sampling and random initialization on the results.

### DRSA network

To assess the effectiveness of the imputation strategy on a deep neural network designed for time-to-event data analysis without competing events, we used the deep recurrent survival analysis (DRSA) architecture by Ren et al.^27^. We picked this architecture because a) it is primarily designed for a single-event discrete-time survival analysis setting and b) because DRSA differs structurally from the DeepHit architecture, therefore, allowing us to assess the effectiveness of the proposed approach with different types of deep neural networks. In contrast to DeepHit that consists of consecutive fully connected layers, DRSA consists of a layer of Long Short-Term Memory (LSTM)^33^ units in addition to fully connected layers. In other words, the DRSA network consists of an initial layer that embeds the input features $x_i$ into a set of vectors. Then through a fully connected layer, the embedded vectors are turned into a middle-representation of the input. The output of this layer is concatenated with the observed time points (*t*) and is fed into the recurrent layer, consisting of a series of LSTM units. In the end, a fully connected layer is used with the Sigmoid activation function to estimate the hazard rates at each time point *t*. For better-calibrated prediction rules and improved discriminatory power, instead of the cross-entropy loss that was used in the original DRSA network, we used the loss function derived from the negative log-likelihood of the discrete time-to-event model^16^. The loss function that was considered for the optimization consisted of two terms $L_l$ and $L_z$, i.e., ${\arg \min }_\theta (1-\alpha )L_{l}(\theta )+\alpha L_{z}(\theta )$, where $\theta$ denotes the set of network parameters, $\alpha$ denotes the tuning parameter balancing the two loss terms, $L_l$ denotes the negative log-likelihood loss and $L_z$ denotes a part of the negative log-likelihood that was only computed for the set of uncensored instances in the training data^16^.

To assess the performance of our proposed method with DRSA, we compared two different setups: (1) *New approach using DRSA with preprocessed input data:* Similar to the experiments with DeepHit, instead of the original input data, we used the modified version of the input data (with $T_i$ replaced by $\vartheta _i$), in which the censoring times corresponding to individuals with observed competing events were imputed using the subdistribution weights. (2) *DRSA that ignores competing events by treating them as censored:* Similar to the first setup, instead of replacing $T_i$ by $\vartheta _i$, we ignored the competing events by treating all individuals with an observed competing event as censored.

Again, each experiment was repeated 10 times per dataset in order to reduce the effect of random sampling and random initialization on the results.

### Data description

In this subsection, we describe the datasets that were used in the experiments. To show the effectiveness of the imputation strategy, we created three sets of simulated competing risks data. Additionally, to test our method in real-world scenarios, we used two datasets from clinical and epidemiological research: The first one was collected for the CRASH-2 clinical trial^19^ mentioned above; the second one was the 2013 breast cancer dataset from the Surveillance, Epidemiology, and End Results (SEER) program^34^.

#### Simulated data

For generating simulated data, we used the discrete model by Berger et al.^35^. Their data generation approach was adopted from Fine and Gray^23^ and Beyersmann et al.^36^, and allowed to create datasets from a discretized subdistribution hazard model with two competing events $\epsilon _i \in \{1,2\}$.

More specifically, Berger et al.^35^ defined their discretized subdistribution hazard model based on the continuous subdistribution hazard model

2 $$\begin{aligned} F_1(t|\varvec{x}_i) \, = \, P(T_{cont,i} \le t, \epsilon _i = 1 \, |\, \varvec{x}_i ) = 1- (1-q+ q \cdot \exp (-t))^{\exp {(\varvec{x}_i^\intercal \varvec{\gamma }_1 })}\text {,} \end{aligned}$$

where $T_{cont,i} \in \mathbb {R}^+$ denotes a continuous time variable and $\varvec{\gamma }_1$ is a set of regression coefficients for individual *i*, with predictor variables $\varvec{x}_i$. We used the parameter *q* to tune the probability of having the event $\epsilon _i = 1$ (defined by $P(\epsilon _i = 1 |\varvec{x}_i) = 1-(1-q)^{\exp {(\varvec{x}_i^\intercal \varvec{\gamma }_i })}$) and the probability of having the competing event $\epsilon _i = 2$ (defined by $P(\epsilon _i = 2 |\varvec{x}_i)= 1 - P(\epsilon _i = 1 |\varvec{x}_i)= (1-q)^{\exp {(\varvec{x}_i^\intercal \varvec{\gamma }_i })}$). Further, the continuous times for the second event were drawn from an exponential model $T_{cont,i}|\epsilon _i = 2 \sim \text {Exp}(\xi _2 = \exp {(\varvec{x}_i^\intercal \varvec{\gamma }_2 }))$, with rate $\xi _2$ and regression parameters $\varvec{\gamma }_2$ for the predictor variables $\varvec{x}_i$. To obtain grouped data, we discretized the continuous event times into $k=20$ time-intervals using empirical quantiles. Analogous to Berger et al.^30^, discrete censoring times were drawn from the probability distribution $P(C_i = t) = {b^{(k+1-t)}}/{\sum _{i=1}^{k} b^i}$, where the parameter $b \in \mathbb {R}^+$ affected the overall censoring rate. Furthermore, we generated four predictor variables: two of them were normally distributed, $x_1, x_2 \sim N(0,1)$, and the other two followed a binomial distribution each, $x_3, x_4, \sim \text {Binomial}(1, 0.5)$. The regression coefficients were the same as in the work by Berger et al.^35^, with $\gamma _1 = c(0.4, -0.4, 0.2, -0.2)^\intercal$ and $\gamma _2 = c(-0.4, 0.4, -0.2, 0.2)^\intercal$. We simulated datasets of size $n=30,000$ with different type-1 event rates $q \in \{0.2, 0.4, 0.8\}$ and a *medium* censoring rate of $b = 1$. In the simulated datasets the empirical censoring rates corresponding to $b=1$ were $\{47.4\%, 47.6\%, 48.0\%\}$, the proportion of type-1 event rates corresponding to values of *q* were $\{11.5\%, 21.8\%, 38.6\%\}$, and consequently type-2 event rates were $\{41.1\%, 30.6\%, 13.4\%\}$.

#### CRASH-2 data

The first real-world dataset used in our experiments was collected for the randomized CRASH-2 (Clinical Randomisation of an Antifibrinolyticin Significant Haemorrhage 2) trial, which was conducted in 274 hospitals in 40 countries between 2005 and 2010^19^. The data provide information on hospital death in adult trauma patients with or at risk of significant haemorrhage. Death was recorded during hospitalization of the patients for up to 28 days after randomization. Up to this date, patients had either died, been discharged alive, transferred to another hospital, or were still alive in hospital. For our analysis we used the publicly available version of the study database at https://hbiostat.org/data/. Based on Table 1 in ^19^, we selected eight variables for analysis: Categorical variables included the sex of the patient (male/female) and type of injury (blunt/penetrating/blunt and penetrating). Continuous and ordinal variables included total Glasgow Coma Score (range 3 to 15, median = 15), the estimated age of the patient (mean = 34.6 years, sd = 14.3 years), number of hours since injury (mean = 2.8, sd = 2.4), systolic blood pressure in mmHg (mean = 97.5, sd = 27.4), respiratory rate per minute (mean = 23.1, sd = 6.7), and heart rate per minute (mean = 104.5, sd = 21.0). After discarding patients with missing values, we analyzed this dataset in two ways: 1) We specified *death due to bleeding* as the event of interest for analysis ($\epsilon = 1$) and considered *discharge from the hospital or death due to other causes* as the competing event ($\epsilon = 2$). In this scenario, the censoring rate is $16.8\%$, the type-1 event rate was $4.9\%$ and the type-2 event rate was $78.3\%$. 2) We specified *death from any cause* as the event for interest for analysis ($\epsilon = 1$) and considered *discharged from the hospital* as the competing event ($\epsilon = 2$). In this scenario, the censoring rate was 16.8, the type-1 event rate was $14.9\%$ and the type-2 event rate was $68.3\%$. Table 1 summarizes the percentage of patients experiencing each event first. These analyses enabled us to investigate the performance of different methods for varying event rates while censoring remained the same.

**Table 1:** Characteristics of the datasets used in the experiments.

Censoring rate	Type-1 rate	Type-2 rate	Training	Validation	Test
**Simulated data**					
$47.4\%$	$11.5\%$	$41.1\%$	15,000	5000	10,000
$47.6\%$	$21.8\%$	$30.6\%$	15,000	5000	10,000
$48.0\%$	$38.6\%$	$13.4\%$	15,000	5000	10,000
**CRASH-2 data**					
$16.8\%$	$4.9\%$	$78.3\%$	9729	3256	6851
$16.8\%$	$14.9\%$	$68.3\%$	9729	3256	6851
**SEER breast cancer data**					
$88.4\%$	$6.9\%$	$4.7\%$	60,898	24,361	36,539

The three leftmost columns represent the censoring, type-1 ($\epsilon = 1$), and type-2 ($\epsilon = 2$) rates in the training/validation/test datasets. The three rightmost columns represent the respective numbers of instances in the simulated, CRASH-2, and SEER breast cancer data. For CRASH-2, $\epsilon = 1$ indicates either death due to bleeding event (upper row) and death due to any recorded cause (lower row).

#### SEER breast cancer data

The second real-world dataset used in our experiments was the 2013 breast cancer data from the Surveillance, Epidemiology, and End Results (SEER) program^34^. Here our focus was on female patients with breast cancer, aged 18-75 years at the time of diagnosis. We specified *patient’s death due to breast cancer* as event of interest ($\epsilon = 1$) and considered *death due to other causes* as the competing event ($\epsilon = 2$). The predictor variables included TNM stage (twelve T stage and four N stage categories), tumor grade (I - IV), estrogen and progesterone receptor statuses (positive/negative), primary tumor site (nine categories), surgery of primary site (yes/no), type of radiation therapy and sequence (seven and six categories, respectively), laterality (right/left), ethnicity (white, black, American Indian/Alaska Native, Asian or Pacific Islander, unknown), Spanish origin (nine categories), and marital status at diagnosis (single, married, separated, divorced, widowed). In addition to these categorical variables, we selected the following continuous and ordinal features; patient’s age at diagnosis (recorded in years, mean age = 55.6 years, standard deviation (sd) = 10.8 years), the number of positive and examined lymph nodes (0-84 and $1, 2, \ldots , 89,$ $90$, respectively), the number of primaries (1-6), and tumor size ($0, 1, \ldots , 988,$ $989$ mm). After discarding patients with missing values, 121, 798 patients remained. For this dataset the censoring rate was $88.4\%$, the type-1 event rate was $6.9\%$ and the type-2 event rate was $4.7\%$. For a detailed explanation of the features, see the SEER text data file description at http://seer.cancer.gov.

### Training setup

*Simulated data.* For our experiments we split the 30,000 instances of each set of simulated data into training ($\mathcal {D}_{train}$) , test ($\mathcal {D}_{test}$) and validation ($\mathcal {D}_{validation}$) sets randomly, making sure that the event and censoring rates were the same across the three datasets. The sizes of the train, test and validation datasets were 15,000, 10,000 and 5000 respectively. Table 1 briefly summarizes the size of the datasets used in each experiment. Since in our method the censoring times for individuals with an observed competing event are randomly imputed, we repeated the experiments 10 times and report the average performance. For each repetition, all of the individuals in training, test, and validation sets remained unchanged, except for the censoring times that were re-imputed.

*CRASH-2 data.* For this dataset, we used the same training setup as for the simulated data. We randomly split the 19, 836 instances into the training, test, and validation sets, using a stratified sampling approach that ensured all had approximately the same censoring and competing event rates (see Table 1). The sizes of the training, test and validation datasets were 9, 729, 6, 851 and 3, 256 respectively.

*SEER data.* We used the same training setup as for the other datasets. We randomly split the 121, 798 instances into the training, test, and validation sets, making sure all had $88.4\%$, $6.9\%$, and $4.7\%$, of censoring, event of interest and competing event rates respectively (see Table 1). The sizes of the training, test and validation datasets were 60, 898, 36, 539 and 24, 361 respectively.

### Evaluation metrics

#### Calibration plots based on the cumulative incidence function (CIF)

To assess the calibration of the fitted models, we performed graphical comparisons of the estimated (model-based) CIF for type-1 events and a respective nonparametric estimate obtained from the Aalen-Johansen method^37^.

Specifically, for input predictor variables $x_i$ from $\mathcal {D}_\text {test}$, the model-based CIF at timepoint *t* for the event of interest was estimated by

3 $$\begin{aligned} \hat{F}_{1}(t|x_i) \, = \, \hat{P}(T\le t, j=1|x_i) \, = \, \sum _{s=1}^{t} \hat{P} (T= s,j=1|x_i)\, , \end{aligned}$$

where the probability estimates $\hat{P}(\cdot )$ in (3) were taken from the output of the DeepHit network (for details, see Lee et al.^9^). Details on the Aalen-Johansen estimator, which is a covariate-free estimator of the CIF, have been given in the book by Klein et al.^37^. In our experiments, we considered a fitted DNN model to be well calibrated if the model-based and covariate-free CIF estimates agreed closely.

#### Concordance index (*C*-index^38,39^)

To evaluate the discriminatory power of each method for the event of interest we used the *C*-index as defined by Wolbers et al.^40^. For a pair of independent individuals *i* and *j* in the $\mathscr {D}_\text {test}$, this measure compares the ranking of a *risk marker* $M(t, x_i)$ at timepoint *t* with the ranking of the survival times of the event of interest. More specifically, summarizing all competing events by $\epsilon = 2$, the *C*-index is defined by

4 $$\begin{aligned} C_{1}(t) \, := \, P\left( M(t,x_{i})>M(t,x_{j}) \, | \, \epsilon _{i}=1\text { and }T_{i}\le t\text { and }(T_{i}<T_{j}\text { or }\epsilon _{j}=2)\right) \, . \end{aligned}$$

In our experiments we defined *M*(*t*, *x*) by the cumulative incidence function (Equation 3). Ideally, the *C*-index takes value 1 if the rankings of the risk marker and the type-1 survival times are in perfect disagreement (i.e., larger marker values are associated with smaller survival times). For our experiments, we used the inverse-probability-weighted estimator by Wolbers et al.^40^ (Equation 4) that is implemented in the R package **pec**.

## Results

**Figure 3:**
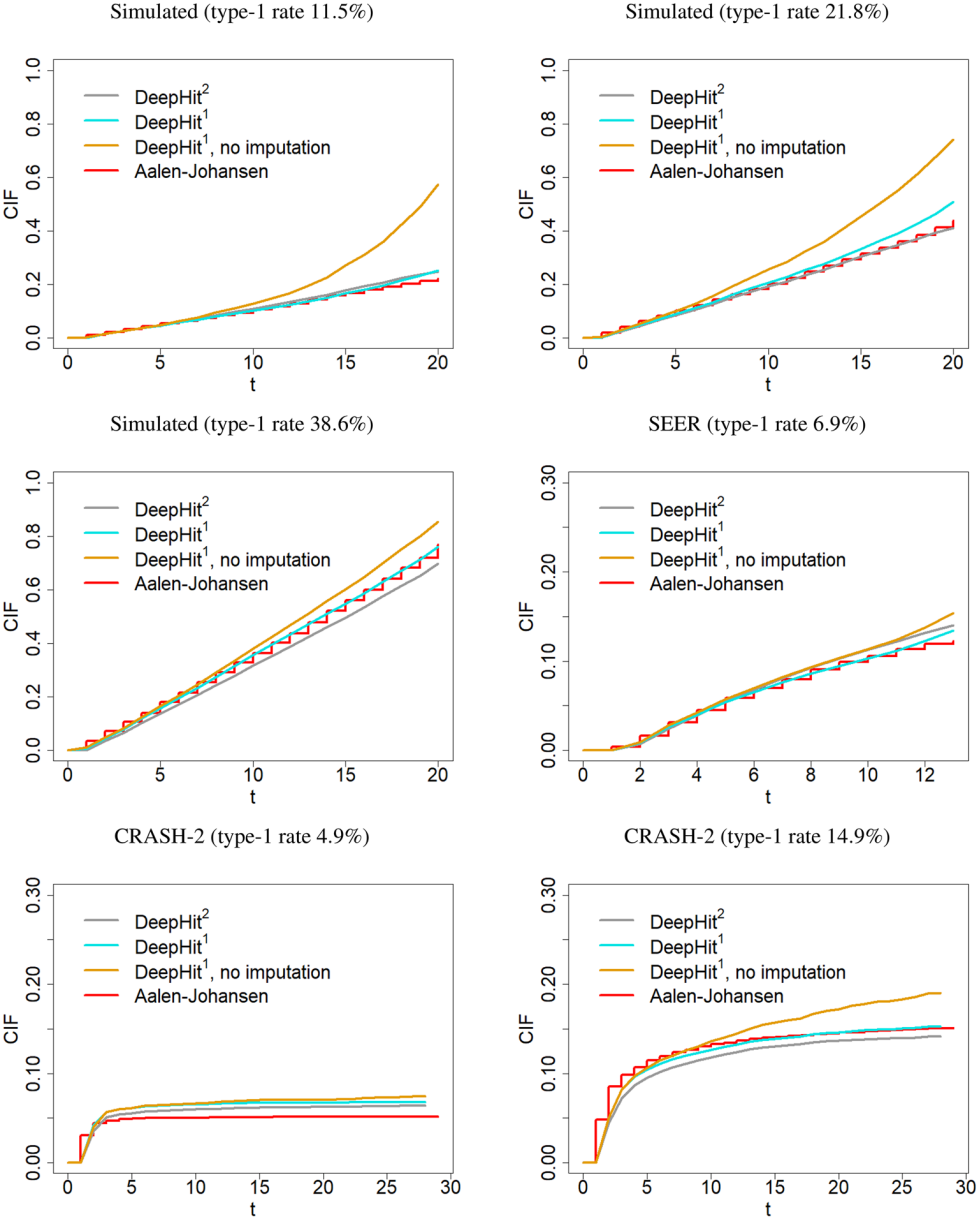
Calibration plots obtained from the test data in Table 1, using the DeepHit architecture. Each plot presents the averaged type-1 cumulative incidence functions as obtained from (i) training the DeepHit$^{1}$ with the preprocessed data (cyan), (ii) training DeepHit$^{1}$ treating individuals with observed competing events as censored (orange), and (iii) training DeepHit$^{2}$ for both the event of interest and the competing event (gray). Red curves refer to the nonparametric Aalen-Johansen reference curves.

The calibration plots for the various model fits are presented in Fig. 3. It is seen that despite the smaller learning capacity of the imputation-based DeepHit$^{1}$ approach, this network resulted in similarly well-calibrated models as the DeepHit$^{2}$ with two sub-networks. Note that in all cases, using the sub-distribution weights for imputing the censoring times led to a better calibration compared to the single-event DeepHit architecture that treated individuals with an observed competing event as censored (thus ignoring the competing events).

Generally, the calibration of the overall average CIF estimate improved with our method when the rate type-1 events became larger. This is seen from the last row of Fig. 3. For the same censoring rates and predictor variables (for CRASH-2), DeepHit$^{2}$ resulted in an underestimation of the CIF when the rate of type-1 events was high. This is also evident in the results from our experiments on simulated data. On the other hand, our proposed method showed an overall less sensitivity to the type-1 event rate. This effect could possibly be due overfitting issues, as adding an additional sub-network for each competing event to the architecture increases the learning capacity of the network without providing enough data to train each pathway.

**Figure 4:**
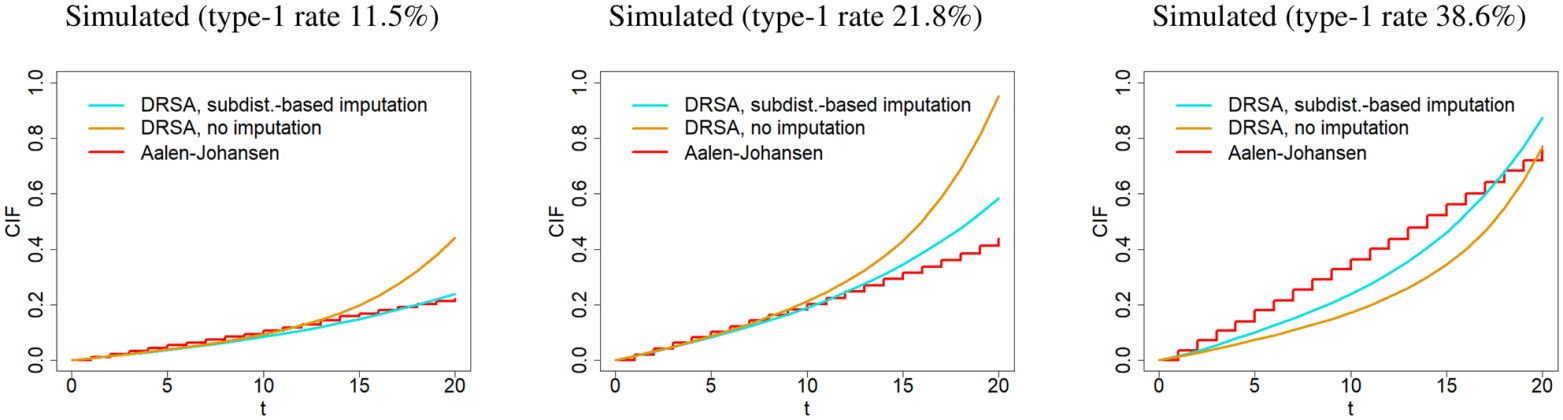
Calibration plots obtained from the simulated test data in Table 1 using the DRSA architecture. Each plot presents the averaged type-1 cumulative incidence functions as obtained from (i) training the DRSA network with the preprocessed training data (cyan) and (ii) training DRSA treating individuals with observed competing events as censored (orange). Red curves refer to the nonparametric Aalen-Johansen reference curves.

The calibration plots for training with DRSA are presented in Fig. 4. It is seen that despite the single-event structure of the DRSA, this network resulted in a well-calibrated model when the type-1 event rate was small. In all cases, using the sub-distribution weights for imputing the censoring times led to a better calibration compared to the experiments that treated individuals with an observed competing event as censored (thus ignoring the competing events). For the same censoring rates and predictive variables, DRSA resulted in an underestimation of the CIF when the rate of type-1 events was high. On the other hand, again our proposed method showed an overall less sensitivity to the type-1 event rate compared to when the competing event was ignored.

Analogous to the results from the calibration plots, the *C*-indices obtained from our imputation-based method showed a discriminatory power that was similar to the respective performance of the other methods (see Table 2). In a number of settings, the discriminatory power even improved when using our method. For instance, in the experiments with the simulated data, the estimated mean *C*-index was highest for the DeepHit$^{1}$ method with imputed censoring times. For CRASH-2 with a type-1 event rate of $4.9\%$ the observed difference ($0.01\%$) between imputation-based DeepHit$^{1}$ and DeepHit$^{2}$ was small. For the type-1 event rate of $14.9\%$ our proposed method performed slightly better. For the SEER breast cancer data, however, DeepHit$^{1}$ without imputation had the best average performance with regard to the *C*-index. This could be due to the fact that the rate of observed competing events was low to the degree that treating the respective event times as censoring times might not have substantially affected the censoring survival function.

**Table 2:** Mean estimated *C*-indices (averaged over time) with estimated standard deviations, as obtained from training the DeepHit architecture on the simulated, CRASH-2, and SEER breast cancer data.

Data	Type-1-rate	Type-2-rate	DeepHit$^{1}$	DeepHit$^{1}$, no imp.	DeepHit$^{2}$
CRASH-2	$4.9\%$	$78.3\%$	78.17 ± 1.04	76.80 ± 4.96	**78.18** ± 0.94
CRASH-2	$14.9\%$	$68.3\%$	**80.14** ± 1.77	79.88 ± 2.01	80.05 ± 4.23
SEER	$6.9\%$	$4.7\%$	81.75 ± 3.46	**81.80** ± 3.49	81.73 ± 3.34
Simulated	$11.5\%$	$41.1\%$	**64.13** ± 0.75	62.58 ± 2.17	63.71 ± 0.96
Simulated	$21.8\%$	$30.6\%$	**65.90** ± 0.69	64.59 ± 2.25	65.20 ± 3.26
Simulated	$38.6\%$	$13.4\%$	**66.05** ± 0.47	64.97 ± 2.51	64.39 ± 6.26

DeepHit$^{1}$ = DeepHit architecture with one sub-network trained with the preprocessed input data; DeepHit$^{2}$ = DeepHit architecture with two subnetworks; DeepHit$^{1}$, no imp. = DeepHit architecture with one sub-network trained on the original input data (treating individuals with observed competing events as censored individuals). Best-performing methods are marked bold. Note that the *C*-indices must be compared within each row, as the datasets used for training were different in terms of size, censoring, and event rates across the rows. For CRASH-2, in the upper and the lower rows $\epsilon = 1$ indicates death due to bleeding and death due to any recorded cause, respectively. The numbers in this table are obtained from the test datasets.

**Table 3:** Mean estimated *C*-indices (averaged over time) with estimated standard deviations, as obtained from training the DRSA architecture on the simulated data.

Data	Type-1-rate	Type-2-rate	DRSA, subdist.-based imp.	DRSA, no imp.
Simulated	$11.5\%$	$41.1\%$	**58.04** ± 0.88	55.62 ± 0.86
Simulated	$21.8\%$	$30.6\%$	**60.10** ± 0.95	57.60 ± 0.93
Simulated	$38.6\%$	$13.4\%$	**64.29** ± 0.93	63.41 ± 1.00

The first column on the right-hand side contains results from DRSA architecture trained with the preprocessed input data; The second column shows the results from the DRSA architecture, trained on the original input data (treating individuals with observed competing events as censored individuals). Best-performing methods are marked bold. Note that the *C*-indices must be compared within each row, as the datasets used for training are different in terms of censoring and event rates across the rows. The numbers in this table are obtained from the test datasets.

Analogous to the experiments with DeepHit, for DRSA, the *C*-indices obtained from our imputation-based method showed an improved discriminatory power compared to the scenario when competing event time was used as censoring (see Table 3). It can be observed that the gap between the performance of our imputation method and ignoring the competing events became smaller with the decrease of type-2 event rate. The reason could be that by the decrease of the observed competing events rate, treating the respective event times as censoring times might not have substantially affected the censoring survival function. Overall, compared to DRSA, DeepHit showed better discriminatory power on the simulated data. Note, however, that systematic performance comparisons of different deep survival architectures are beyond the scope of this work.

In terms of execution time, we observed that the average time needed for training the deep networks reduced by $21\%$ for the simulated data, $10\%$ for the SEER, and $37\%$ for the CRASH-2 dataset using our method. This time reduction is possibly due to the reduced number of parameters involved in the training of DeepHit$^{1}$ compared to DeepHit$^{2}$ (see Table 4). Consequently, in applications with more than one competing event, where three or more subnetworks are added to the architecture, the decrease in computation time when using our algorithm is expected to be even greater. The average number of iterations, however, was on the same order of magnitude for both DeepHit$^{1}$ and DeepHit$^{2}$. For all datasets on average DeepHit$^{1}$ took 15, 022 iterations and DeepHit$^{2}$ 15, 277. Note that the stopping criterion for all of the networks was the performance on the validation data.

**Table 4:** Average time (in seconds) and number of iterations needed for training DeepHit$^{1}$ and DeepHit$^{2}$ per dataset.

	Simulated	SEER	CRASH-2
	Time | #itr	Time | #itr	Time | #itr
DeepHit$^{1}$	$184.78$ | 10, 666	$827.97$ | 22, 600	$116.47$ | 11, 800
DeepHit$^{2}$	$235.32$ | 9133	$918.39$ | 22, 300	$185.30$ | 14, 400

Performance on validation data was used as the stopping criterion.

## Discussion

Even though deep neural networks are increasingly used for survival analysis, it is still relatively complicated to adapt the available methodology to situations with competing events. This is in contrast to the classical statistical literature, in which a wide variety of methods are available^20–23,41^, and in which it is widely agreed that competing-risks analyses are often necessary to avoid biased estimation results and/or predictions^36^. Although several adaptations to DNN architectures have been proposed recently^9,11,24^, these adaptions rely on a huge number of parameters, making network training and regularization a challenging task. In this work, we showed that an imputation strategy based on subdistribution weights could convert the competing risks survival data into a dataset that is specifically tailored to analyzing the event of interest only. This conversion enables the use of any of the much simpler deep survival network architectures that are designed to handle a single event of interest in the presence of right censoring. Our experiments on simulated and real-world datasets illustrated that this preprocessing step not only simplifies the training in terms of number of parameters and running time but also preservers the accuracy in terms of discriminatory power and calibration. The method could be further stabilized by implementing a multiple imputation approach (analogous to the continuous-time method by Ruan and Gray^32^); however, such an approach would dramatically increase the run time and would be infeasible in the context of training DNN architectures. Further, in our experiments we observed that multiple imputations did not have a major effect on predictive performance in our datasets containing several thousands of instances with event rates larger than $\sim 5\%$. Our codes for simulated data generation, censoring time imputation, and the experiments are available at https://github.com/shekoufeh/Deep-Survival-Analysis-With-Competing-Events.
